# Aucuboside Inhibits the Generation of Th17 Cells in Mice Colitis

**DOI:** 10.3389/fphar.2021.696599

**Published:** 2021-07-16

**Authors:** Chenxue Mei, Xiao Wang, Fanxiang Meng, Xiaoqing Zhang, Ling Chen, Siqi Yan, Junxiu Xue, Xun Sun, Yuanyuan Wang

**Affiliations:** ^1^ Department of Immunology, College of Basic Medical Sciences of China Medical University, Shenyang, China; ^2^ Department of Gastroenterology, Shengjing Hospital of China Medical University, Shenyang, China; ^3^ Department of Gastroenterology, Jinqiu Hospital of Liaoning Province, Shenyang, China; ^4^ Department of Anesthesiology, The Fourth Affiliated Hospital, China Medical University, Shenyang, China

**Keywords:** aucuboside, inflammation, Treg, Th17, colitis

## Abstract

Aucuboside is an iridoid glycoside extracted from traditional Chinese medicine such as *Rehmannia glutinosa*, possessing a wide range of biological activities, including antioxidant, anti-aging, anti-inflammatory, and anti-fibrotic effects. The effects of aucuboside on inflammatory bowel disease (IBD) have not been studied. Therefore, the effects of aucuboside on the generation of Foxp3+ regulatory T (Treg) cells and IL-17–producing T helper (Th17) cells in colitis were studied. A mouse colitis model was established by intracolonic administration of 2,4,6-trinitrobenzene sulfonic acid (TNBS) to mimic human IBD. The generation of Treg and Th17 cells was evaluated by flow cytometry. Aucuboside significantly alleviated colitis symptoms, including weight loss, high disease activity index, and inflammatory responses. The generation of Th17 cells in colitis was significantly inhibited by aucuboside and accompanied by the suppression of IL-17 expression. In Raw264.7 cells, the LPS-induced increase in IL-17 expression was also suppressed by aucuboside, which was significantly blocked by the RORγt inhibitor sr2211. In addition, the decrease in the proportion of Treg cells was also partially reversed by aucuboside, which may reflect the aucuboside-induced inhibition of Th17 cells. This previously unrecognized immunoregulatory function of aucuboside may have clinical applications in IBD.

## Introduction

Inflammatory bowel disease (IBD) is an inflammatory disease of the colon with multifactorial etiology, including ulcerative colitis (UC) and Crohn’s disease (CD). IBD is characterized by periodic remission and deterioration (McDowell and Haseeb, 2019). The incidence of IBD worldwide varies between 0.5 and 24.5 per 100,000 people, while in China, the occurrence rate of UC is 11.6 and that of CD is 1.4 per 100,000 people (Sugimoto et al., 2008). IBD promotes significant gastrointestinal symptoms, such as bloody diarrhea, abdominal pain, anemia, and weight loss (Mateus et al., 2018; Siracusa et al., 2020). Fibrosis, obstruction, and cancer are complications of IBD that often affect the quality of life of patients (Zhang et al., 2021). Current medical therapies such as corticosteroids and immunomodulators are still limited by some severe side effects and complications. Therefore, it is still necessary to discover some more novel pharmacologic approaches.

The pathogenesis of IBD is multifactorial, with increasing evidence suggesting an important role in the balance between regulatory T cells (Treg) and Type 17 T helper cells (Th17) (Geremia et al., 2014). Th17, a subtype of CD4+ T cells, are pro-inflammatory T helper cells that play a dominant role in the production of pro-inflammatory cytokines, such as interleukin (IL)-17, IL-21, and IL-22, which serve as markers of inflammation (Nakae et al., 2003). Excessive IL-17 secreted by Th17 would recruit neutrophils and monocytes and increase the production of TNF-α and IL-1β, contributing to tissue damage and exacerbating inflammation in the gut (Kolls and Lindén, 2004; Korn et al., 2009). Another subtype of CD4+ T cells, Tregs, are immunosuppressive cells that participate in the regulation of colitis progression (Cao et al., 2004). Intercellular interactions and the secretion of IL-10 are the main pathways by which Tregs can regulate the inflammatory response (Rothstein and Camirand, 2015).

It is factually beneficial to uncover potential drugs/compounds to regulate the generation of Tregs in the treatment of IBD. Zhang et al. firstly reported that D-mannose, a C-2 epimer of glucose, stimulates Treg cell differentiation in human and mouse cells. Both aucuboside and D-mannose can be isolated from *Lathraea squamaria* L. (Swiatek and Dombrowicz, 1976); however, the effects of aucuboside on the generation of Tregs/Th17 cells were not studied. This study aimed to explore the effects of aucuboside on the generation of Tregs/Th17 cells in colitis.

Aucuboside used in this study was isolated from the traditional Chinese medicinal plant *Eucommia ulmoides*. *Eucommia ulmoides* exerts a wide range of biological activities including antioxidant, anti-aging, anti-inflammatory, and anti-fibrotic effects (Murakami et al., 2018). In this study, the effects of aucuboside on Treg/Th17 balance and aucuboside-induced Treg/Th17 balance in colitis were investigated. A mouse colitis model was established using intracolonic administration of 2,4,6-trinitrobenzene sulfonic acid (TNBS) to mimic human IBD.

## Materials and Methods

### Reagents

Aucuboside (purity ≥98%) was obtained from Chengdu Pusi Biotechnology Co., Ltd. (Chengdu, China). Sulfasalazine (SASP) was purchased from Tianjin Kingyork Group Co., Ltd. (Tianjin, China). Antibodies to iNOS (ab110304), p-p65 (ab62484), cleaved caspase 3 (cl-caspase3, ab13847), Foxp3 (ab21685), and RORγt (ab59348) were purchased from Abcam (Hong Kong) Ltd. (Hong Kong, China). Chemicals were obtained from Sigma-Aldrich (St. Louis, MO, United States) unless otherwise indicated.

### Animals and Experiment Design

The experimental protocol was approved by the China Medical University Animal Care and Ethics Committee (CMU2019218), SYXK (Liao) 2018-0008. Thirty male SPF C57BL/6 mice of 6–8 weeks of age were acclimatized to laboratory conditions (23°C, 12 h/12 h light/dark, 50% humidity, and ad libitum access to food and water) for 2 weeks before the experiments. The animal protocol was designed to minimize pain and discomfort to the animals. Mice were housed one per cage and were deprived of food for 12 h before the experiments.

After adaptive feeding for one week, 60 mice were randomly divided into six groups (*n* = 6/group): group I, sham-operated control with intracolonic administration of saline; group Ⅱ, colitis group; and groups Ⅲ, IV, and V treated with SASP (100 mg/kg body weight, intragastric, dissolved in saline), low-dose aucuboside (20 mg/kg body weight, intragastric, dissolved in saline), and high-dose aucuboside (80 mg/kg body weight, intragastric, dissolved in saline), respectively, 1 day after the induction of colitis. SASP and aucuboside were administered by gavage once per day for 14 consecutive days. The mice colitis model was induced as described previously (Xiong et al., 2016; Mateus et al., 2018). Briefly, mice were fasted for 24 h with free access to drinking water. A catheter was inserted through the anus to the approximate level of the splenic flexure under urethane anesthesia. The colon was then infused with 100 μL TNBS dissolved in ethanol (50% v/v). The mice were allowed to eat and drink ad libitum from 1 h after the operation. Distal colon samples from the full-thickness intestinal wall were harvested for biochemical studies. After 14 days, blood samples were collected from the eye, and then mice were sacrificed by cervical dislocation. The spleen and mesenteric lymph nodes were collected, and colon tissue samples were retained and preserved. Body weight, food intake, and defecation were recorded daily. The disease activity index and the colon weight/length ratio were determined. Colon histology, morphology, and the severity of inflammation were assessed in hematoxylin and eosin (HE)-stained colon sections. Harvested colon tissue specimens were cut into 5 mm pieces, processed, and mounted on slides for immunohistochemical and western blot analyses.

### Treg/Th17 Analysis by Flow Cytometry

Fresh spleen and mesenteric lymph nodes were placed in 3 ml PBS containing 10% FBS and ground for 2 min on a 200-mesh nylon sieve using a 5 ml syringe piston and transferred to 15 ml EP tubes. The spleen tissue homogenate was centrifuged at 1,100 r/min for 10 min, and the deposited cells were retained. RBC lysate (2 ml; Beijing Solarbio Science & Technology Co., Ltd., Beijing, China) was added and incubated under dark conditions for 15 min and then centrifuged at 2,240 r/min for 5 min. After washing with 10 ml RPMI-1640 solution, a single lymphocyte cell suspension was obtained. The mesenteric lymph node homogenate was centrifuged at 2,000 r/min for 5 min; the deposited cells were retained; and after washing with 10 ml RPMI-1640 solution, a single lymphocyte cell suspension was obtained. The prepared single-cell suspension was transferred to a 1.5 ml EP tube by secondary filtration using a 200-mesh nylon sieve, and the cell number was adjusted to 1 × 10^6^/ml.

To detect the number of Tregs, collected cells were incubated with 2 µL pre-chilled anti-mouse CD4 and CD25 antibodies (BD Biosciences, United States) and fixed at 4°C in a dark room for 30 min. After washing with PBS containing 10% FBS and centrifuging at 1,200 r/min for 6 min at 4°C, cell membrane breaking lysate (BD Biosciences) was added to the cell suspension, avoiding light, and incubated for hours. Then, 2 µL pre-chilled anti-mouse factor Foxp3 antibody (BD Biosciences) was added to the collected cells and fixed at 4°C in a dark room for 30 min. To detect the number of Th17 cells, PMA (30 ng/ml), ionomycin (1 μg/ml), and BFA (10 ng/ml) were added to the collected cells and incubated for 4 h before filtering and adjusting the cell number. The staining of Th17 cells using FITC anti-mouse CD4 and APC anti-mouse IL-17A followed the same procedure as Tregs. After washing, the cells were resuspended in 200 µL PBS containing 10% FBS for flow cytometry analysis.

### Quantitative Real-Time Polymerase Chain Reaction

RNA isolation was carried out using Trizol (Takara, Dalian, China) per the manufacturer’s instructions. Total RNA was quantified using a NanoDrop instrument to measure optical density (OD) at 260/230 nm, and purity was evaluated by obtaining the absorbance ratio at 260/280 nm. cDNA was synthesized from 1 μg of total RNA with a Thermo Scientific Verso cDNA Kit according to the manufacturer’s instructions. Quantitative real-time PCR (qRT-PCR) was performed using 10–20 ng of cDNA with SYBR green master mix (Takara, United States) and a StepOnePlus RT-PCR system (Applied Biosystems, Inc., United States). The differences in mRNA expression at all the gene levels were calculated as the fold change using the formula 2-ΔΔct. Primer sequences for Foxp3 were as follows: Foxp3 forward: 5′-TGG​CTC​CAA​GGA​TGG​TTA​GC-3′ and Foxp3 reverse: 5′- TCA​GGG​ACA​GGG​TTG​ACA​GT-3′ (provided by General Biosystems). Primer sequences for RORγT were as follows: RORγT forward: 5′-TCC​ATA​TTT​GAC​TTT​TCC​CAC​T-3′ and RORγT reverse: 5′-GAT​GTT​CCA​CTC​TCC​TCT​TCT​C-3′. As a control, mRNA content for GAPDH was analyzed using the following primers: GAPDH forward: 5′-GCC​ACC​CAG​AAG​ACT​GTG​GAT-3′ and GAPDH reverse: 5′-GGA​AGG​CCA​TGC​CAG​TGA-3′ (provided by General Biosystems).

### Statistical Analysis

GraphPad Prism (version 9.0; GraphPad Prism Software, Inc., La Jolla, CA, United States) was used to perform statistical analysis. A non-parametric test, one-way ANOVA, was used for data comparisons between three or more groups, followed by the Kruskal–Wallis rank-sum test. All results were obtained from at least three independent experiments.

## Results

### Effects of Aucuboside on Normal Mice

**FIGURE 1:**
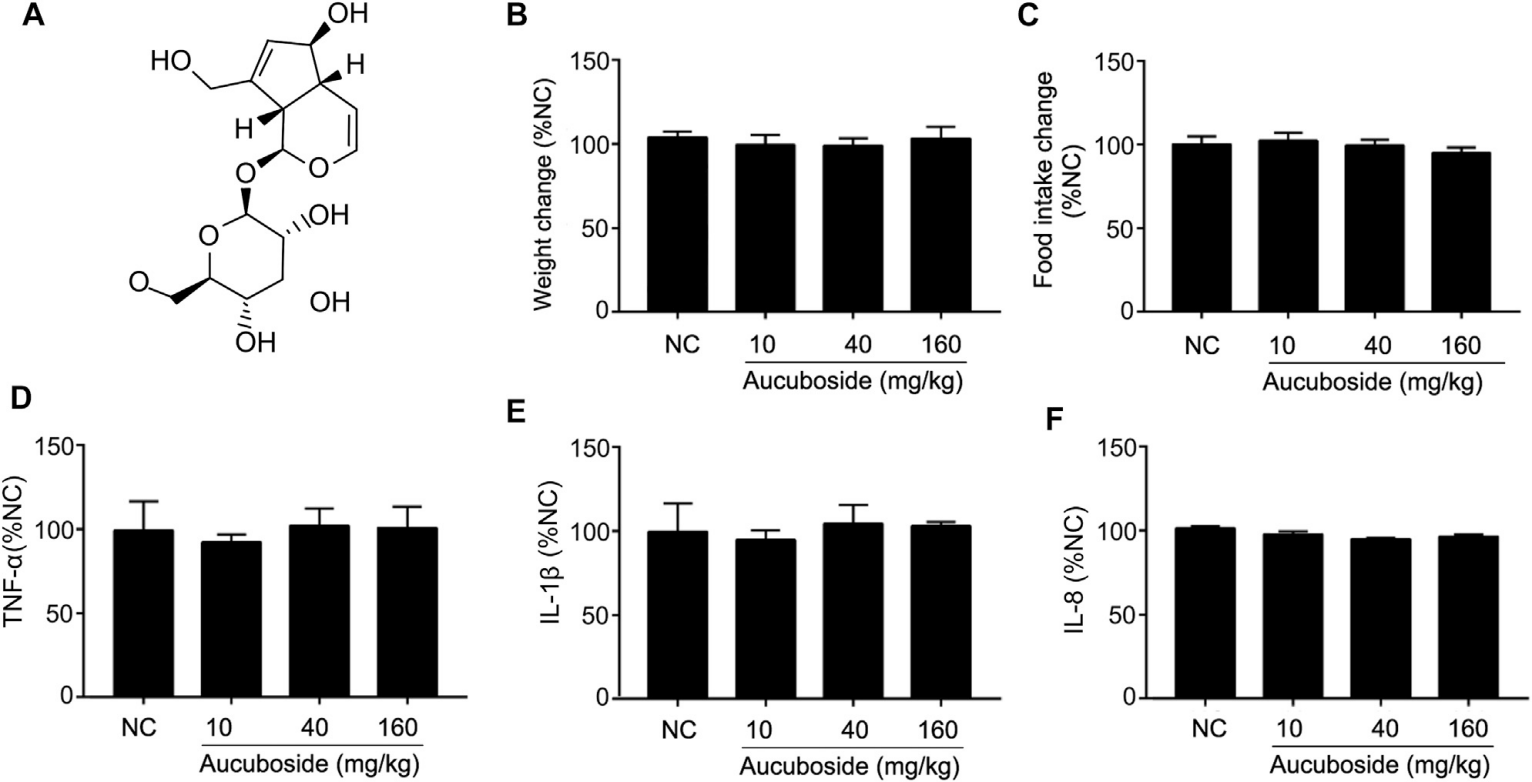
Effects of aucuboside on normal mice, toxicology of arbutin *in vitro* and *in vivo*, and chemical structure of aucuboside **(A)**. Effects of gavage administration of aucuboside on body weight change **(B)**, food intake **(C)**, and colonic expression of TNF-α, IL-1β, and IL-8 **(D–F)**. Data are expressed as mean ± SD. Values in the normal control (NC) group are 100%, and other data are given relative to the NC value.

The chemical structure of aucuboside is shown in Figure 1A. For normal control mice, the gavage administration of 10, 40, and 160 mg/kg aucuboside consecutively for 14 days did not significantly affect body weight (Figure 1B), food intake (Figure 1C), or colonic pro-inflammatory cytokines, including TNF-α (Figure 1D), IL-1β (Figure 1E), and IL-8 (Figure 1F). Based on these preliminary experiments and previous reports, we selected the maximum dose of aucuboside as 80 mg/kg in this study.

### Aucuboside Ameliorated Colitis Symptoms

**FIGURE 2:**
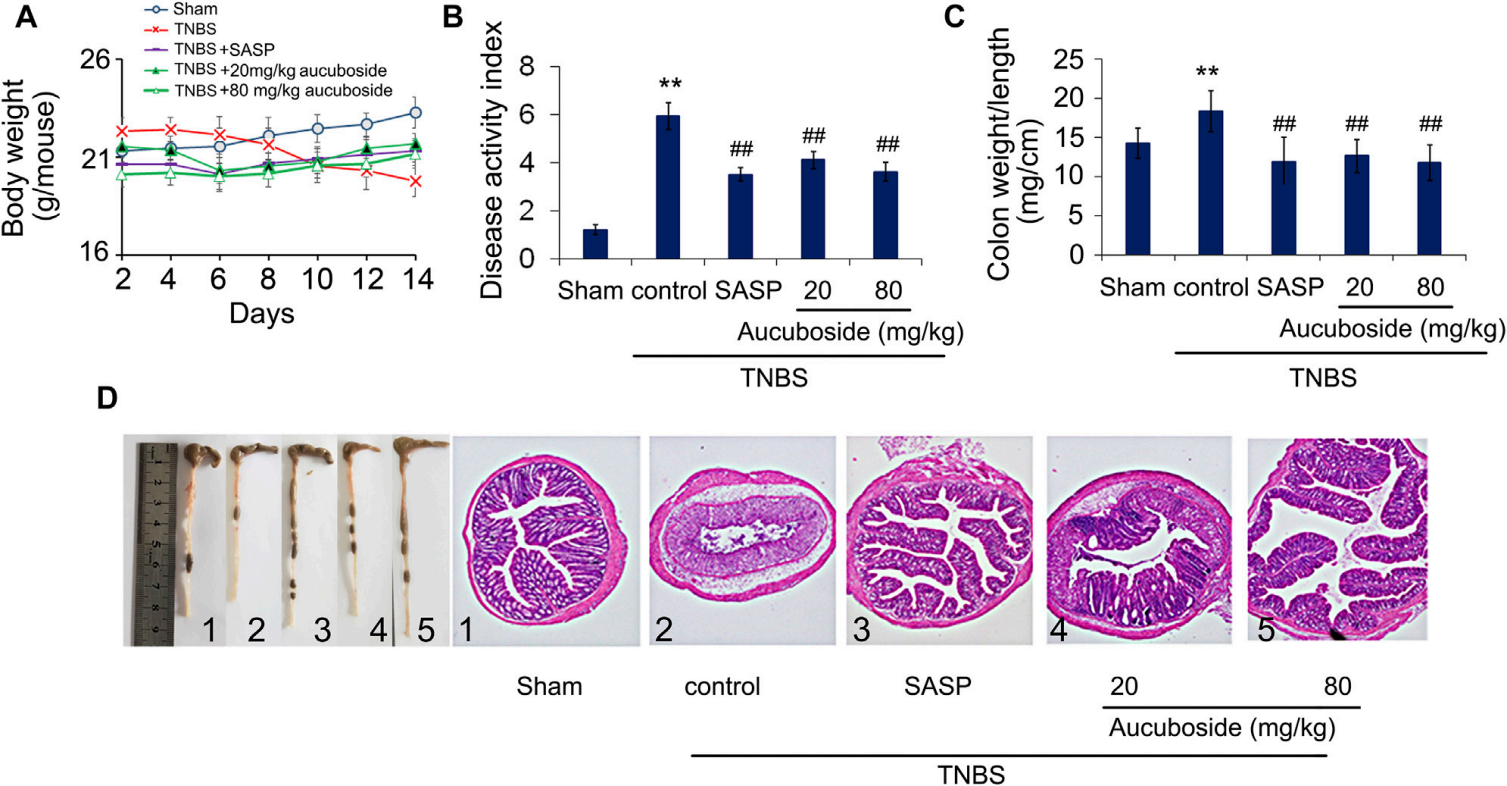
Effects of aucuboside on colitis symptoms. After 13 days of treatment, the effects of aucuboside on body weight loss **(A)**, disease activity index **(B)**, and colon weight-to-length ratio **(C)** were evaluated. The macroscopic observation and hematoxylin–eosin–stained colon sections (scale bar = 100 μm) **(D)** are also shown. Data are expressed as mean ± SD. Values in the sham group are 100%, and other data are given relative to the sham group. **p < 0.01 compared with the sham group; ##*p* < 0.01 compared to the colitis control; *n* = 6 mice.

Compared with the sham group, body weight loss (Figure 2A), higher disease activity index (Figure 2B), and an increased colon weight-to-length ratio (Figure 2C) were observed in the TNBS colitis group. The micromorphology and histomorphology are shown in Figure 2D. Compared with the sham control, destroyed microvilli, incomplete mucosal structure, and infiltration of immune cells were all significant. Similar to the SASP-induced treatment effects, all pathological changes in colitis were significantly reversed by aucuboside.

### Aucuboside Alleviated Cytokine Profile in Colitis

**FIGURE 3:**
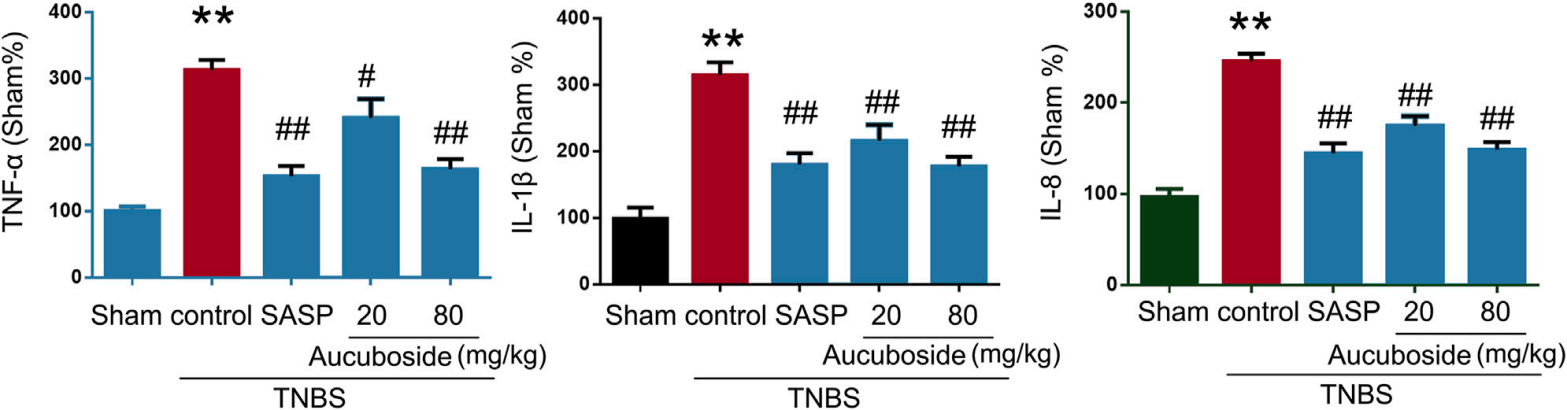
Effects of aucuboside on cytokine expression. After 13 days of aucuboside treatment, the colonic expressions of TNF-α, IL-1β, and IL-8 **(A–C)** were measured. Data are expressed as mean ± SD. **p < 0.01 compared with the sham group; ^#^ *p* < 0.05, ^##^ *p* < 0.01 compared with the colitis control; *n* = 6 mice.

Compared with the sham group, colonic pro-inflammatory cytokines, including TNF-α, IL-1β, and IL-8, were significantly increased in the colitis group, and these changes could be significantly reversed by 20 and 80 mg/kg aucuboside (Figure 3). These results showed that aucuboside could decrease inflammation in colitis.

### Aucuboside Reduced the Release of Inflammatory Mediators and Inhibited Apoptosis

**FIGURE 4:**
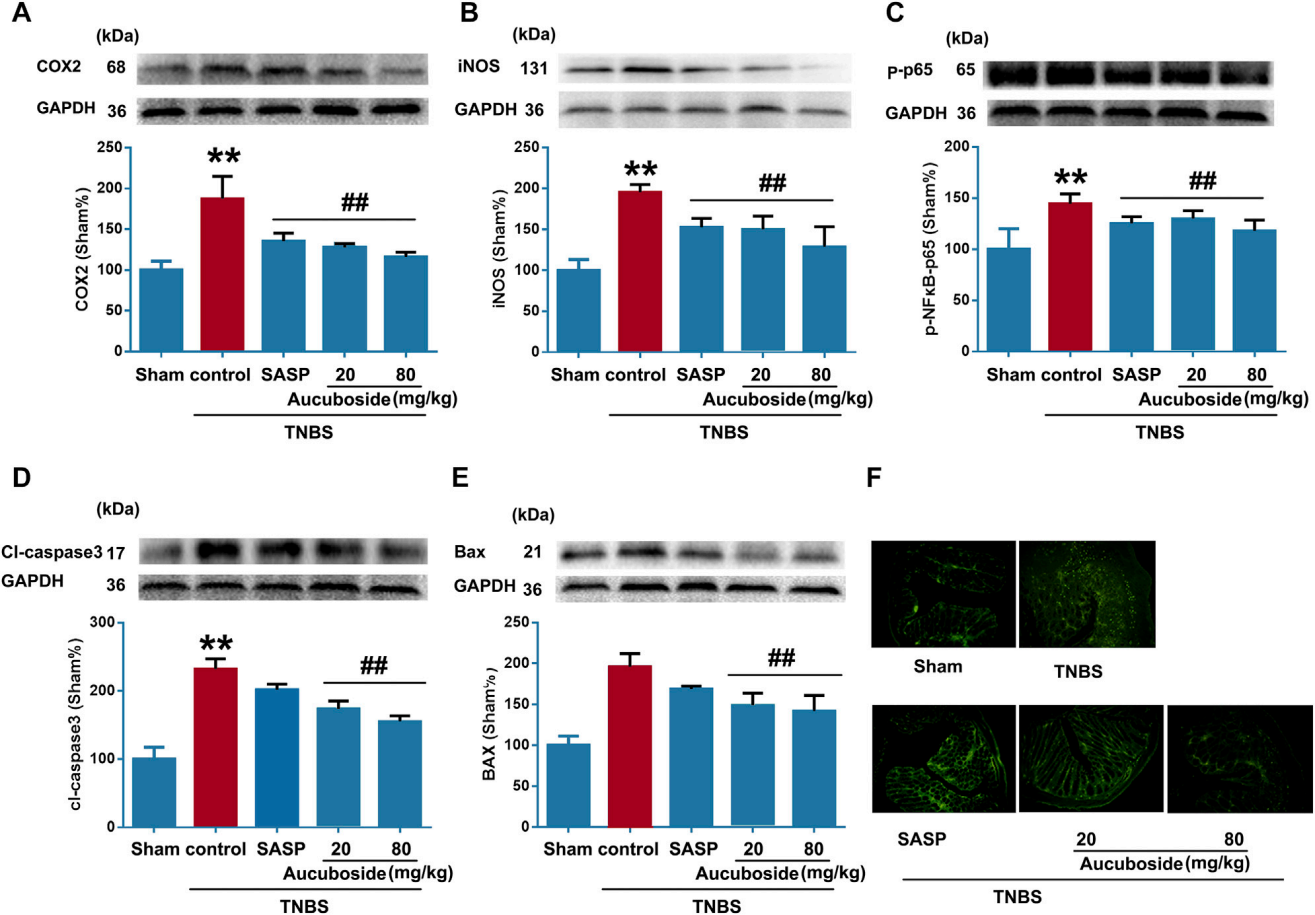
Effects of aucuboside on inflammation and apoptosis in colitis. After 13 days of aucuboside treatment, effects on colonic inflammatory mediators **(A)** COX-2, **(B)** iNOS, and **(C)** phosphorylated NF-κB-p65 (p-p65) were studied by western blotting. The effects of aucuboside on apoptosis-related proteins **(D)** cleaved caspase 3 (cl-caspase3) and **(E)** Bax were studied by western blotting. **(F)** TUNEL was used to test the effects of aucuboside on epithelial cell apoptosis in colitis. Data are expressed as mean ± SD. Values in the sham group are 100%, and other data are given relative to the sham group. **p < 0.01 compared with the sham group; ^##^ *p* < 0.01 compared with the colitis control; *n* = 6 mice.

As shown in Figures 4A–C, colonic epithelial COX-2, iNOS, and phosphorylated NF-κB-p65 (p-p65) were significantly increased compared with the sham control; both SASP and aucuboside significantly reversed these changes. These results further confirmed that aucuboside could exert anti-inflammation effects on colitis.

The apoptosis markers cl-caspase3 (Figure 4D) and Bax (Figure 4E) were significantly increased in the colitis group, which was reversed by 20 and 80 mg/kg aucuboside. The TUNEL-positive cells in the colonic epithelium of the colitis group were significantly increased compared with the sham group. After treatment with 20 and 80 mg/kg aucuboside, the TUNEL-positive cells in the colonic epithelium were significantly decreased (Figure 4F). These results suggested that aucuboside has anti-apoptotic effects.

### Effects of Aucuboside on the Generation of Th17 Cells

**FIGURE 5:**
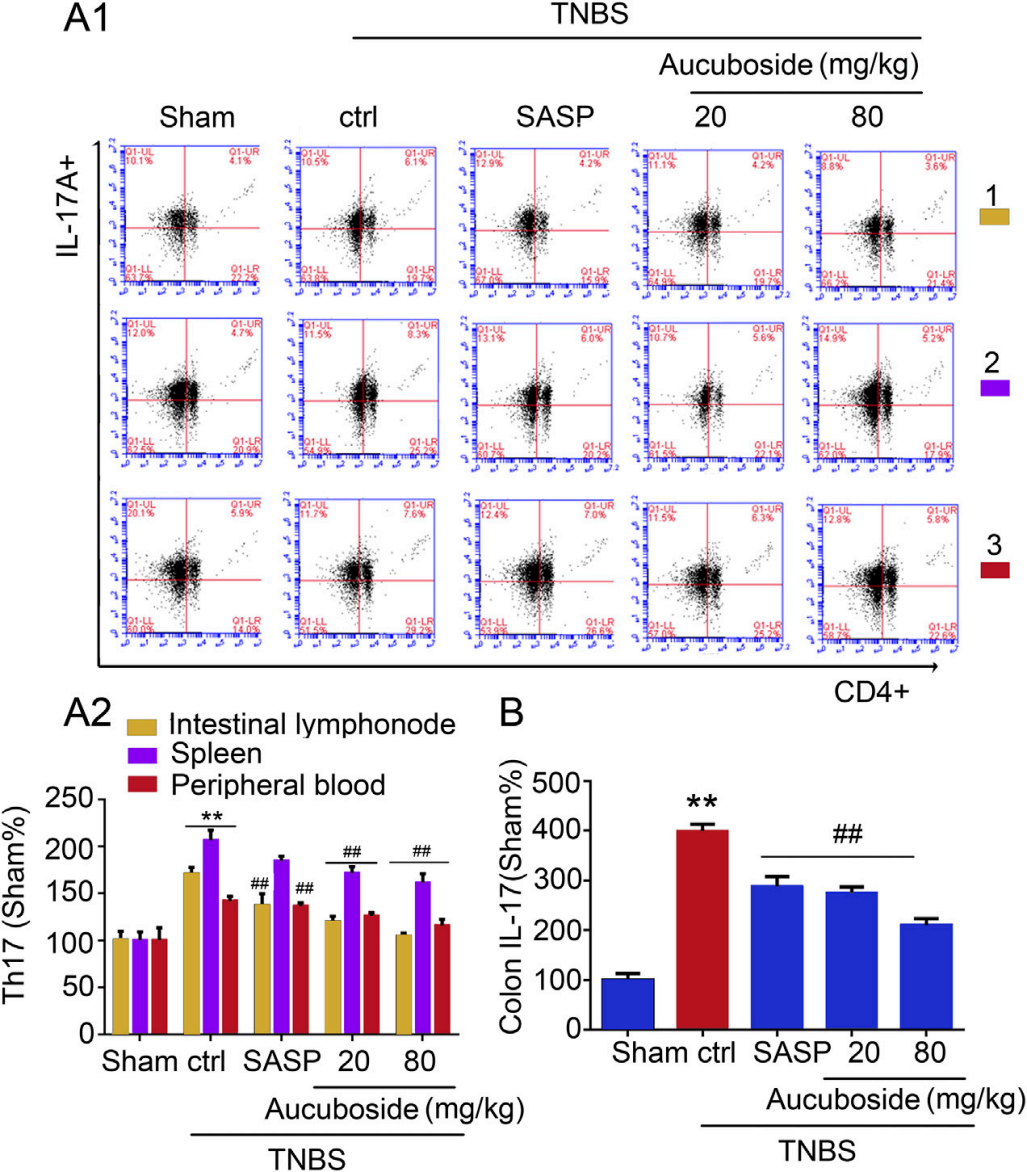
Effects of aucuboside on the generation of Th17 cells. **(A) (i)** and **(ii)** are the representative image and statistical analysis of the generation of Th17 cells in colonic mesenteric lymph nodes (1), spleen (2), and peripheral blood (3), respectively. **(B)** Effects of aucuboside on release of IL-17. Values in the sham group are 100%, and other data are given relative to the sham group. **p < 0.01 compared with the sham group; ^##^ *p* < 0.01 compared with the colitis control; *n* = 6 mice.

Compared with the sham group, the generation of Th17 cells in colonic mesenteric lymph nodes, spleen, and peripheral blood was significantly increased in the colitis group (Figures 5Ai,ii). Gavage administration of 20 and 80 mg/kg aucuboside significantly decreased the proportion of Th17 in the colitis group (Figures 5Ai,ii). IL-17, as an effective cytokine in Th17 cells, was also studied in this study. As shown in Figure 5B, IL-17 was significantly increased in the colitis group, which was significantly reversed by different doses of aucuboside. These results suggest that the inhibition of Th17 production may be involved in the effects on colitis induced by aucuboside.

### Effects of Aucuboside on the Generation of Treg Cells

**FIGURE 6:**
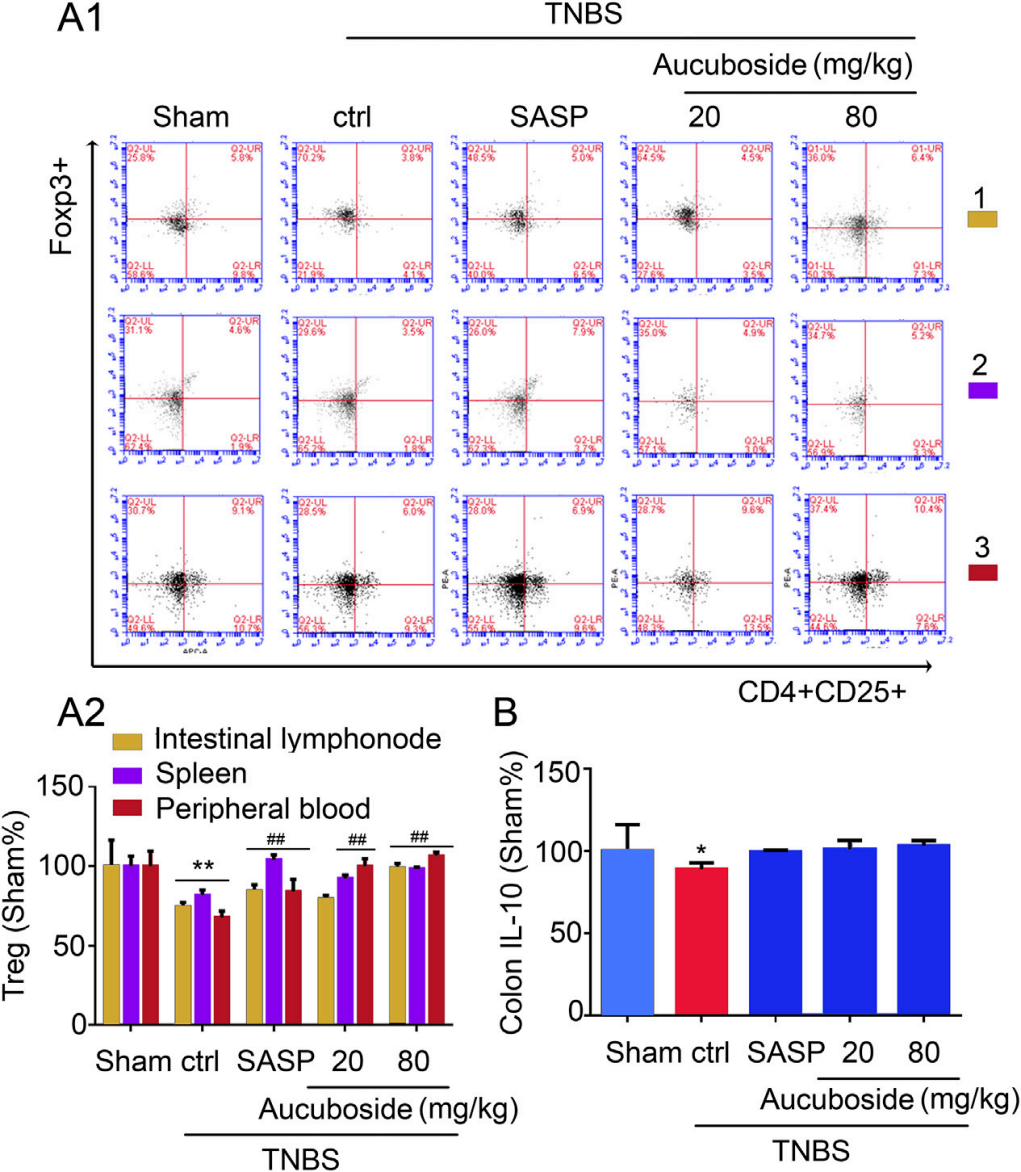
Effects of aucuboside on the generation of Treg cells. **(A) (i)** and **(ii)** are the representative image and statistical analysis of the generation of Treg cells in colonic mesenteric lymph nodes (1), spleen (2), and peripheral blood (3), respectively. **(B)** Effects of aucuboside on release of IL-10. Values in the sham group are 100%, and other data are given relative to the sham group. *p < 0.05, **p < 0.01 compared with the sham group; ^##^ *p* < 0.01 compared with the colitis control; *n* = 6 mice.

Compared with the sham group, the Treg cell number in colonic mesenteric lymph nodes, spleen, and peripheral blood was significantly decreased in the colitis group (Figures 6Ai,ii). Gavage administration of 20 and 80 mg/kg aucuboside significantly increased the Treg cell number in the colitis group (Figures 6Ai,ii). However, IL-10, the effective cytokine of Tregs, was not significantly affected by aucuboside (Figure 6B). These results suggest that an increase in Tregs may not be the main mechanism associated with aucuboside treatment.

### Mechanisms Underlying Aucuboside-Induced Inhibition of Th17

**FIGURE 7:**
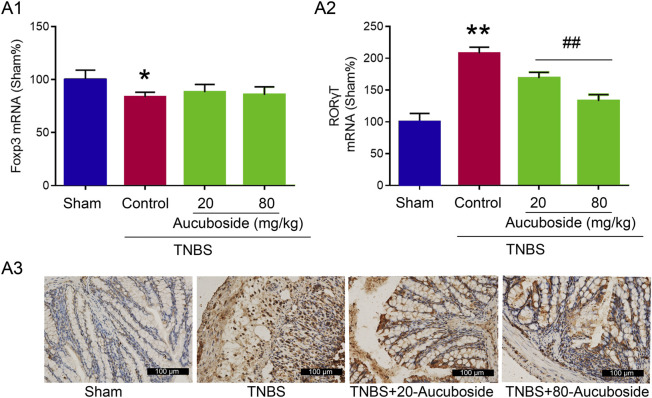
Potential mechanism underlying aucuboside-induced regulation of Th17 cells and Treg cells. **(A) (i)** Effects of aucuboside on Foxp3 mRNA expression and **(ii)** effects of aucuboside on RORγt mRNA expression; **(iii)** immunohistochemical analysis of RORγt expression. *p < 0.05, **p < 0.01 compared with sham; ^##^ *p* < 0.01 compared with the colitis control; *n* = 6 independent experiments.

To clarify the mechanisms underlying the aucuboside-induced regulation of Treg and Th17 cell generation, the mRNAs of Foxp3 and RORγt were studied. As shown in Figure 7Ai, the colonic Foxp3 mRNA was significantly decreased in the colitis group, which was not significantly affected by 20 and 80 mg/kg aucuboside. Colonic RORγt, as the main regulating factor promoting Th17 cell differentiation, was significantly increased in colitis, and the increase was reversed by both 20 and 80 mg/kg aucuboside (Figures 7Aii,iii). These results suggest that aucuboside may inhibit Th17 cell generation through blocking RORγt activity.

## Discussion

In this study, the effects of aucuboside and underlying mechanisms on mice colitis were studied. Gavage administration of aucuboside significantly alleviated TNBS-induced colitis; the colitis symptoms including weight loss, higher disease activity index, mucosal necrosis, and inflammatory cell infiltration were significantly alleviated by aucuboside. The generation of Th17 cells in colitis was inhibited by aucuboside. The inhibition of Th17 cells is the mechanism involved in the aucuboside-induced treatment effect in colitis.

The expression of inflammatory cytokines and inflammatory mediators in colitis was significantly reduced by aucuboside. These results suggested that aucuboside has an anti-inflammation function. It was also shown that aucuboside can inhibit inflammatory responses in experimental traumatic brain injury (Wang et al., 2020). Our study indicated that aucuboside has an anti-inflammatory potential in colitis. Aucuboside administration also significantly inhibited intestinal epithelial cell apoptosis in colitis. All these results suggested that aucuboside can exert protective effects against TNBS-induced colitis in mice.

We focused on the effects of aucuboside on the generation of Treg and Th17 cells because the increase in Th17/Treg is one of the main pathological mechanisms of colitis. Our study showed that the number of Th17 cells in colitis was reduced by aucuboside and the IL-17 expression was also suppressed by aucuboside. Aucuboside-induced inhibition of Th17 is related to the blocking of RORγt. RORγt is a major transcription factor that regulates Th17 cell differentiation, and RORγt inhibitors were effective in reducing the severity of experimental autoimmune diseases (Ivanov et al., 2007; Bassolas-Molina et al., 2018). Aucuboside also reversed the decrease in Treg cell number in colitis, but this did not affect the anti-inflammation function. We speculated that the aucuboside-induced activation of Tregs could be a reflection of Th17 inhibition. Further experiments confirmed our supposition because aucuboside did not affect the expression of Foxp3. In the infiltration of inflammatory cells caused by IBD, CD4+ T cells are closely concerned with disease activity and disease progression and associated with pro-inflammatory cytokine levels (Műzes et al., 2012; Smids et al., 2017). The balance of Tregs/Th17 cells is one of the important mechanisms of IBD progression. In the initial differentiation stage, CD4+ T cells are activated by the synergistic effect of TGF-β and IL-6 and RORγt activation is simultaneously induced, leading to the final differentiation into Th17 cells (Bettelli et al., 2006; Sandquist and Kolls, 2018). RORγt can also regulate the function of mature Th17 cells, which is necessary for IL-17 generation (Ivanov et al., 2006). In IBD patients, the over-activation of Th17 cells mediated by RORγt and impaired Treg function is often observed, which leads to a break in the balance of Treg and Th17 cells (Yamada et al., 2016; Hou and Bishu, 2020). The extract of traditional Chinese medicine *Eucommia* can downregulate various pro-inflammatory cytokines, including IL-17, to treat rheumatoid arthritis in rats (Wang et al., 2018). In our experiments, aucuboside extracted from *Eucommia* could reverse the increase of pro-inflammatory cytokines caused by inflammatory cell infiltration in a colitis model. Aucuboside improves the Treg/Th17 balance and finally leads to the alleviation of TNBS-induced colitis.

There are also some shortcomings in this study. First, the detailed underlying mechanisms of how aucuboside inhibits Th17 differentiation were not studied. According to our current results, it is proper that aucuboside may inhibit the secretion of specific cytokines which inhibits RORγt activity, which finally results in the inhibition of the generation of Th17 cells. However, the detailed mechanism needs further study. Second, the binding pattern and interaction mode between aucuboside and RORγt were not elaborated clearly. However, these shortcomings did not affect our main findings. Aucuboside is significantly protective against TNBS-induced colitis; moreover, Th17 inhibition is one of the important mechanisms.

In conclusion, aucuboside can not only alleviate the damaging effects caused by inflammatory cell infiltration in a mouse colitis model but also maintain the balance between Th17 and Treg cells. Further studies are also needed to clarify the detailed mechanisms of how aucuboside inhibits Th17 differentiation in colitis. The results of our study provide a scientific basis for the treatment of IBD using aucuboside and also indicate that aucuboside is one of the main active ingredients of some traditional medicine such as *Rehmannia glutinosa* in IBD treatment.

## Data Availability

The original contributions presented in the study are included in the article/Supplementary Material, and further inquiries can be directed to the corresponding author.
