# Supplementary figures and images for: Aucuboside Inhibits the Generation of Th17 Cells in Mice Colitis
Source: Front Pharmacol. 2021 Jul 16;12:696599. doi: 10.3389/fphar.2021.696599 (PMC8322701; doi:10.3389/fphar.2021.696599)

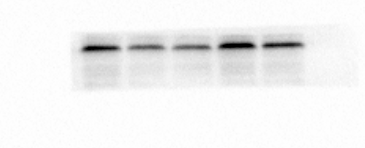

Supplement: Supplementary file 1 [file DataSheet1.ZIP › WB/BCL2/BCL2 1.tif]

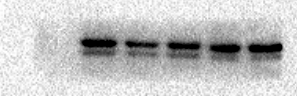

Supplement: Supplementary file 1 [file DataSheet1.ZIP › WB/BCL2/BCL2 2.tif]

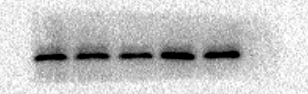

Supplement: Supplementary file 1 [file DataSheet1.ZIP › WB/BCL2/BCL2 3.tif]

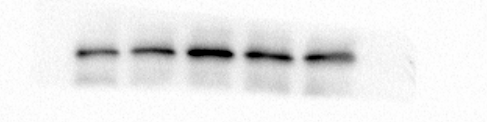

Supplement: Supplementary file 1 [file DataSheet1.ZIP › WB/bcl2-内参/1.png]

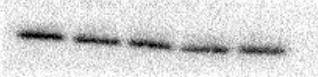

Supplement: Supplementary file 1 [file DataSheet1.ZIP › WB/bcl2-内参/3.jpg]

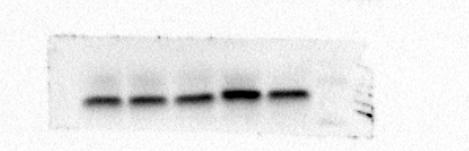

Supplement: Supplementary file 1 [file DataSheet1.ZIP › WB/bcl2-内参/GAPDH B1.tif]

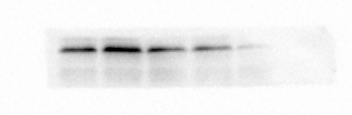

Supplement: Supplementary file 1 [file DataSheet1.ZIP › WB/COX2/COX2 1.tif]

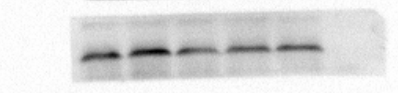

Supplement: Supplementary file 1 [file DataSheet1.ZIP › WB/COX2/COX2 2.tif]

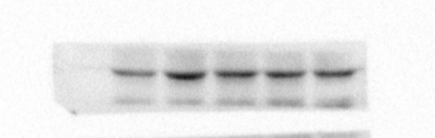

Supplement: Supplementary file 1 [file DataSheet1.ZIP › WB/COX2/COX2 3.tif]

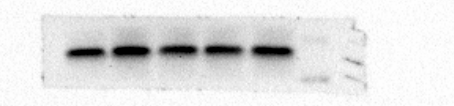

Supplement: Supplementary file 1 [file DataSheet1.ZIP › WB/cox2内参/GAPDH 1.tif]

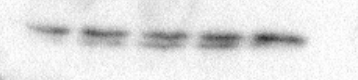

Supplement: Supplementary file 1 [file DataSheet1.ZIP › WB/cox2内参/gapdh 2.png]

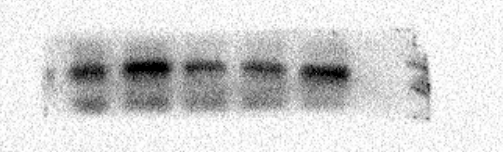

Supplement: Supplementary file 1 [file DataSheet1.ZIP › WB/cox2内参/gapdh 3.png]

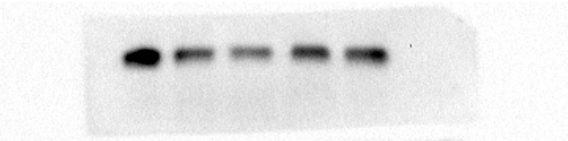

Supplement: Supplementary file 1 [file DataSheet1.ZIP › WB/foxp/1..tif]

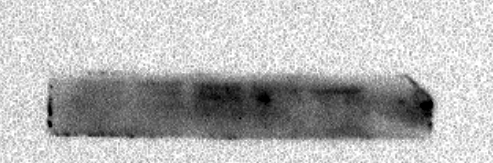

Supplement: Supplementary file 1 [file DataSheet1.ZIP › WB/foxp/2..tif]

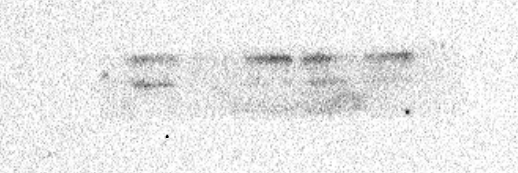

Supplement: Supplementary file 1 [file DataSheet1.ZIP › WB/foxp/3..tif]

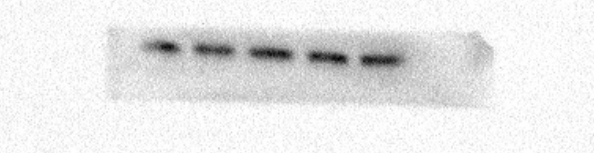

Supplement: Supplementary file 1 [file DataSheet1.ZIP › WB/FoxP3内参/1.tif]

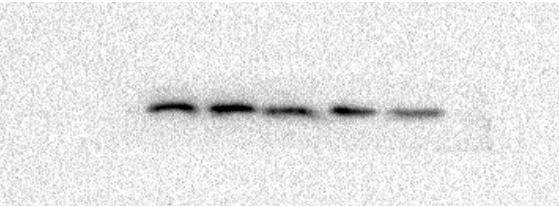

Supplement: Supplementary file 1 [file DataSheet1.ZIP › WB/FoxP3内参/2.tif]

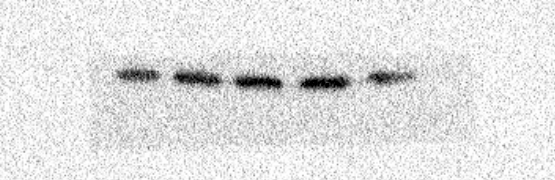

Supplement: Supplementary file 1 [file DataSheet1.ZIP › WB/FoxP3内参/3.tif]

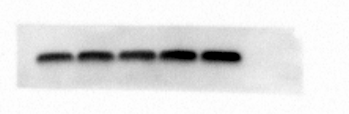

Supplement: Supplementary file 1 [file DataSheet1.ZIP › WB/INOS/GAPDH.tif]

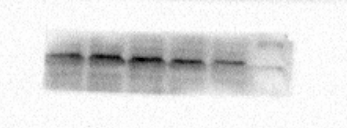

Supplement: Supplementary file 1 [file DataSheet1.ZIP › WB/INOS/INOS1.tif]

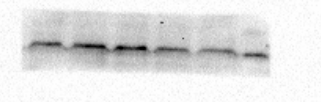

Supplement: Supplementary file 1 [file DataSheet1.ZIP › WB/INOS/INOS2.tif]

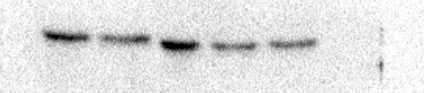

Supplement: Supplementary file 1 [file DataSheet1.ZIP › WB/iNOS内参/1.png]

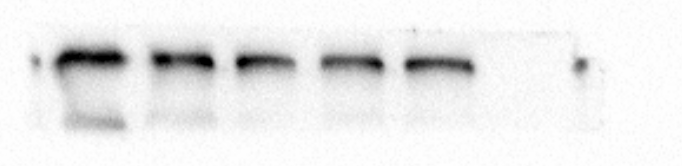

Supplement: Supplementary file 1 [file DataSheet1.ZIP › WB/iNOS内参/2.png]

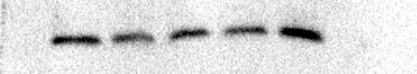

Supplement: Supplementary file 1 [file DataSheet1.ZIP › WB/iNOS内参/3.png]

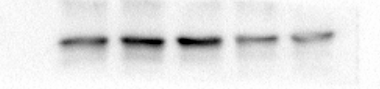

Supplement: Supplementary file 1 [file DataSheet1.ZIP › WB/ror/2.png]

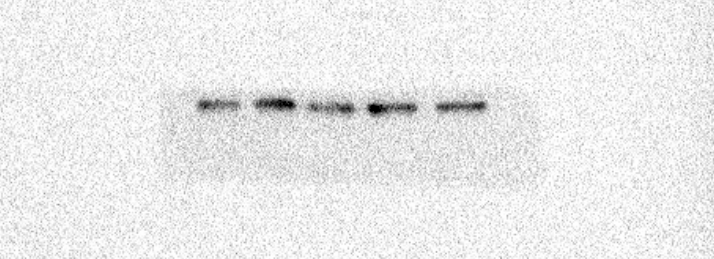

Supplement: Supplementary file 1 [file DataSheet1.ZIP › WB/ror/3.tif]

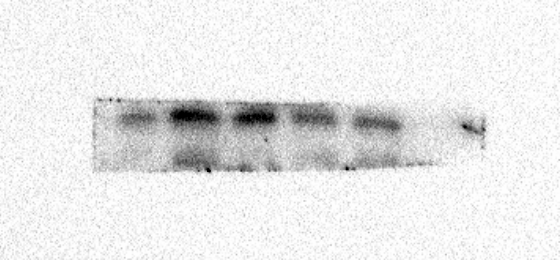

Supplement: Supplementary file 1 [file DataSheet1.ZIP › WB/ror/ror2.tif]

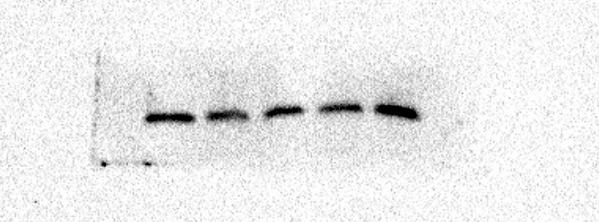

Supplement: Supplementary file 1 [file DataSheet1.ZIP › WB/ROR内参/ga.tif]

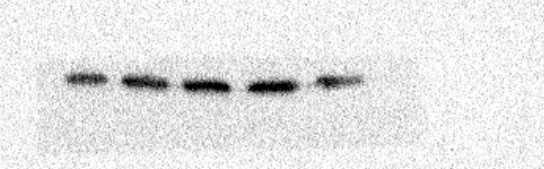

Supplement: Supplementary file 1 [file DataSheet1.ZIP › WB/ROR内参/gapdh 1.tif]

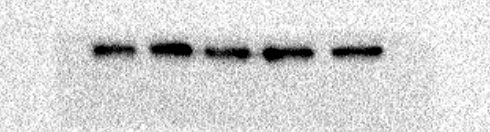

Supplement: Supplementary file 1 [file DataSheet1.ZIP › WB/ROR内参/gapdh2.tif]
